# Improvements in HIV treatment outcomes among indigenous and non-indigenous people who use illicit drugs in a Canadian setting

**DOI:** 10.7448/IAS.19.1.20617

**Published:** 2016-04-18

**Authors:** M-J Milloy, Alexandra King, Thomas Kerr, Evan Adams, Hasina Samji, Silvia Guillemi, Evan Wood, Julio Montaner

**Affiliations:** 1 British Columbia Centre for Excellence in HIV/AIDS, St. Paul's Hospital, Vancouver, BC, Canada; 2 Division of AIDS, Department of Medicine, University of British Columbia, Vancouver, BC, Canada; 3 Faculty of Health Sciences, Simon Fraser University, Burnaby, BC, Canada; 4 Canadian Institutes of Health Research HIV Trials Network, Vancouver, BC, Canada; 5 First Nations Health Authority, West Vancouver, BC, Canada; 6 School of Population and Public Health, University of British Columbia, Vancouver, BC, Canada

**Keywords:** HIV, AIDS, indigenous, highly active antiretroviral therapy, HAART, plasma HIV-1 RNA viral load, treatment-as-prevention

## Abstract

**Introduction:**

In many settings worldwide, members of indigenous groups experience a disproportionate burden of HIV. In Canada, there is an urgent need to improve HIV treatment outcomes for indigenous people living with HIV (IPLWH), to not only reduce HIV/AIDS-associated morbidity and mortality but also curb elevated rates of viral transmission. Thus, by comparing indigenous and non-indigenous participants in an ongoing longitudinal cohort of HIV-positive people who use illicit drugs, we sought to investigate longitudinal changes in three HIV treatment indicators for IPLWH who use illicit drugs during a community-wide treatment-as-prevention (TasP) initiative in British Columbia, Canada.

**Methods:**

We used data from the ACCESS study, an ongoing observational prospective cohort of HIV-positive illicit drug users recruited from community settings in Vancouver, British Columbia. Cohort data are linked to comprehensive retrospective and prospective clinical records in a setting of no-cost HIV/AIDS treatment and care. We used multivariable generalized estimating equations (GEE) to evaluate longitudinal changes in the proportion of participants with exposure to antiretroviral therapy (ART) in the previous 180 days, optimal adherence to ART (i.e. ≥95% vs. <95%) and non-detectable HIV-1 RNA viral load (VL <50 copies/mL plasma).

**Results:**

Between 2005 and 2014, 845 individuals were recruited, including 326 (39%) self-reporting any indigenous ancestry, and contributed 6732 interviews and 13,495 VL measurements. Among indigenous participants, the proportion with recent ART increased from 51 to 94% and non-detectable VL from 23 to 65%. In multivariable models, later interview period was positively associated with recent ART (adjusted odds ratio (AOR)=1.16 per interview period, 95% confidence interval (CI): 1.11 to 1.20) and non-detectable VL (AOR=1.07, 95% CI: 1.04 to 1.10). In adjusted models comparing indigenous and non-indigenous participants, we did not observe differences between the two groups (all *p*>0.1).

**Conclusions:**

In this large and long-term study involving community-recruited HIV-positive illicit drug users, we observed a substantial and increasing proportion of indigenous participants reach several important thresholds in HIV care at rates indistinguishable from non-indigenous participants. The current findings highlight the important role of TasP on vulnerable populations in this setting and contribute to the evidence base supporting the immediate scale-up of ART to address HIV/AIDS-associated morbidity, mortality and viral transmission.

## Introduction

In many settings and among many groups, antiretroviral therapy (ART) has led to dramatic decreases in HIV/AIDS-associated morbidity and mortality among people living with HIV (PLWH) [1,2]. In addition, it is now well established that ART-induced reductions on plasma HIV-1 RNA viral load (VL) markedly reduce HIV transmission [3,4], Thus, a renewed enthusiasm has emerged to scale up HIV treatment as a means of preventing HIV/AIDS-related morbidity and mortality and, in addition, curbing the incidence of new infections. These treatment-as-prevention (TasP) initiatives seek to identify individuals living with HIV infection and promptly engage them in HIV/AIDS treatment and care, especially members of marginalized and vulnerable groups [4,5].

It is estimated there are approximately 400 million indigenous people worldwide, living on all inhabited continents [6,7]. In general, they experience poorer health than analogous non-indigenous groups, most often owing to the results of colonization, dispossession and marginalization [6,7]. Disproportionate burdens of HIV/AIDS have been described amongst many indigenous groups in the United States [8], Asia-Pacific [9] and Latin America [10]. In Canada, there is large cultural, linguistic, economic and political diversity among indigenous groups. However, indigenous peoples, especially First Nations women and those who use illicit drugs, experience significantly higher levels of HIV infection and less favourable treatment outcomes [6–11]. Although it can be problematic to generalize both within and amongst indigenous groups [7], studies of HIV risk in North America have identified a range of proximate risk factors, including injection drug use and sex work, themselves rooted in the ongoing effects of colonization [12,13]. Similarly, all evidence presented to date has described a persistent deficit in treatment access among indigenous people living with HIV (IPLWH), including lower levels of effective ART and higher rates of mortality, compared with non-indigenous PLWHA [14–16]. Thus, evidence of more effective interventions and health systems improvements to reduce elevated levels of preventable HIV/AIDS-related morbidity and mortality among IPLWH is clearly needed.

Over a decade ago, clinicians, policymakers and service providers in Vancouver, British Columbia, began implementing efforts to expand access and support adherence to ART among members of marginalized and vulnerable groups, through means such as simplified treatment algorithms, maximally assisted treatment programmes and harm-reduction-based out-patient centres, as part of an informal effort to promote TasP [4,17–19]. In 2010, the Government of British Columbia formally initiated a TasP-based campaign to expand highly active antiretroviral therapy (HAART) use and engagement in HIV/AIDS treatment and care, especially among traditionally under-treated groups including people who use illicit drugs, women engaged in sex work and IPLWH [20]. Although findings from a province-wide evaluation have described sustained reductions in province-wide VL and significant declines in the rate of new HIV infections [4], long-term trends in HIV treatment patterns and related outcomes among indigenous individuals who use drugs have not been reported. Thus, in the present study, we seek to evaluate trends in key HIV treatment outcomes over time both among IPLWH and between IPLWH and non-indigenous PLWH within the context of a community-wide TasP initiative in British Columbia, Canada.

## Methods

For these analyses, we used data from the AIDS Care Cohort to evaluate Exposure to Survival Services (ACCESS), an observational prospective cohort of HIV-positive illicit drug users. The study has been described in detail previously [21,22]. Briefly, we used community-based strategies including word-of-mouth, postering and snowball sampling to recruit HIV-positive illicit drug users in Vancouver's Downtown Eastside (DTES) neighbourhood, an area with high levels of illicit drug use, poverty and homelessness. Individuals were eligible for inclusion if they are HIV-seropositive as demonstrated by serology, aged ≥18 years and had used illicit drugs other than cannabis in the 30 days prior to the baseline interview. The ACCESS study has been approved by the University of British Columbia/Providence Healthcare Research Ethics Board. All participants provided written informed consent.

Following study recruitment, all ACCESS participants complete an interviewer-administered survey that elicits information on lifetime and recent characteristics, behaviours and exposures. They also undergo an examination by a study nurse, including the provision of a blood sample for analysis. At recruitment, individuals provide their personal health number (PHN), a unique and persistent identifier issued for medical billing and tracking purposes to all residents of British Columbia. Using this identifier, study staff established a confidential linkage with the British Columbia Centre for Excellence in HIV/AIDS (BC-CfE) Drug Treatment Programme (DTP). Through the DTP, the BC-CfE provides HIV/AIDS treatment and care including all medications and clinical monitoring to all individuals living with HIV in BC through the province's no-cost universal medical system. A complete retrospective and prospective clinical profile is available for all ACCESS participants through this linkage. This profile includes all plasma VL observations and CD4 cell counts conducted through the study or as a part of ongoing clinical care, if any. In addition, this linkage contains records from the DTP's pharmacy, the province's sole source of HAART, including data on antiretroviral agent, dose and date dispensed for all HAART-exposed participants. Following the first interview, each participant completes a follow-up interview and nurse examination every 180 days. In these analyses, each interview period contains all the study interviews completed in specific and discrete 180-day calendar periods (e.g. interview period 1 was from 1 December 2005 to 30 May 2006; period 2 was from 1 June 2006 to 30 November 2006). Each individual could only be interviewed once in any interview period.

For the current analyses, we included all individuals recruited between 1 November 2005 and 30 May 2014, who completed ≥1 study interview and had ≥1 plasma VL observation within ±360 days of their baseline interview. Among these participants, we included all baseline and follow-up interviews completed during the study period.

In these analyses, the outcomes of interest were three measures of engagement in the HIV cascade of care: recent dispensation of HAART (yes vs. no), ≥95 adherence to prescribed HAART (yes vs. no) and plasma VL <50 copies/mL plasma (yes vs. no). We ascertained each outcome measure using information contained within each participant's comprehensive retrospective and prospective clinical monitoring records held at the BC-CfE. VL data were augmented by the results of serologic analysis of blood samples collected at each interview. For each period, we determined recent dispensation of HAART if there was a pharmacy record indicating the individual had picked up ≥1 day of HAART in the 180 days prior to the interview.

As in previous research, we defined optimal adherence as ≥95% adherence to HAART in the previous 180 days. We calculated adherence as the ratio of the number of days of HAART dispensed in pharmacy records during the period to the number of days since an individual had initiated HAART, to a maximum of 180 days. We have previously demonstrated this validated measure of pharmacy refill data is strongly associated with plasma VL non-detectability and survival. Finally, for each period, we defined VL as the mean of all observations within the 180-day period prior to the interview dichotomized at <50 versus ≥50 plasma HIV RNA copies/mL. (The Roche Amplicor Monitor assay (Roche Molecular Systems, Pleasanton, California, USA) was used to determine plasma VL from participant blood samples.) If there were no observations within the 180-day period prior to the interview, we declared that an individual had a detectable plasma VL unless they were HAART-exposed and pharmacy refill data indicated >95% adherence for the entire period.

To compare treatment outcomes between indigenous and non-indigenous participants, our primary explanatory variable of interest was self-reported indigenous ancestry (yes vs. no). This variable was based on the baseline question: “What ethnic group or family background do you identify with?” All individuals who replied “First Nations,” “Aboriginal,” “Inuit,” “Métis” or reported any identification or affiliation with any specific indigenous group in Canada or the United States (e.g. “Mohawk” and “Cree”) were defined to have indigenous ancestry. To adjust for any underlying clinical differences between the indigenous and non-indigenous participants, we also considered a number of additional covariates including self-reported gender (male vs. non-male), age at baseline (per year older) and CD4 cell count (per 100 cells/mL). CD4+ cell count was time-updated and determined in the same manner as plasma VL. However, if any 180-day period lacked ≥1 CD4 observations, we used the most recent observation. We also included a covariate describing the interview period (e.g. November 2005 to May 2006; June 2006 to October 2006).

As a first step, we compared socio-demographic and clinical characteristics of all participants at the baseline interview, stratified by indigenous ancestry. Next, to visualize changes in the outcomes of interest over time, we constructed plots of the proportion of indigenous and non-indigenous individuals satisfying each outcome of interest at each six-month interview period over calendar time. Our third step was to quantitatively test for differences in the proportion of indigenous and non-indigenous participants achieving each treatment threshold using generalized estimating equation (GEE) modelling. For each outcome, we estimated its relationship with indigenous ancestry and the other explanatory variables in bivariable models. Next, we fit multivariable models, each including one outcome of interest and all covariates.

To assess possible changes in treatment outcomes among indigenous participants over time, we used the interview period as the primary explanatory variable. As above, we used bivariable and multivariable GEE models restricted to observations from indigenous participants to quantitatively assess the likelihood of experiencing each positive treatment outcome over time. To adjust for possible differences among indigenous participants, we included age, gender and CD4+ cell count in all three multivariable models.

## Results

Between November 2005 and May 2013, 845 HIV-seropositive individuals were recruited, completed ≥1 study interview and had ≥1 plasma VL measurement within ±180 days of their baseline interview and were included in these analyses. Over the study period, the participants contributed 6732 interviews, or a median of 8 (interquartile range (IQR)=4 to 12) per participant, equal to 3366 person-years of follow-up. During this observation time, the participants completed 13,495 VL tests, or a median of 2 (IQR=1 to 3) per interview period. Of the 6732 180-day periods, 744 (11%) did not contain a VL test. This proportion did not differ by indigenous status (11 vs. 11%, *p*=0.943).

The socio-demographic and clinical characteristics of the participants at baseline stratified by self-reported indigenous ancestry are reported in Table 1. Of note, indigenous participants were younger (41 vs. 44 years, *p*<0.001), more likely to be non-male (47 vs. 26%, *p*<0.001), with lower CD4+ cell counts at baseline (340 vs. 380 cells/mL, *p*=0.002). A similar proportion (72%, *p*=0.881) of participants had ever been dispensed ART at baseline and in the previous 180 days (60 vs. 61%, *p*=0.869). Although a significantly lower proportion of IPLWH attained ≥95% adherence (46 vs. 58%, *p*=0.004), the prevalence of plasma VL non-detectability was indistinguishable (29 vs. 33%, *p*=0.216) at baseline.

**Table 1:** Baseline characteristics of 845 HIV-positive illicit drugs users stratified by indigenous ancestry in Vancouver, Canada

Characteristics	Non-indigenous ancestry 519 (61.4) *n* (%)	Indigenous ancestry 326 (38.6) *n* (%)	OR^a^	95% CI^b^	*p*
Age					
Median (IQR)	44 (37 to 50)	41 (34 to 46)	0.96	0.94 to 0.97	<0.001
Gender					
Non-male	133 (25.6)	152 (46.6)	1.00		
Male	386 (74.4)	174 (53.4)	0.39	0.29 to 0.53	<0.001
CD4+ cell (per 100 cells/mL)					
Median (IQR)	3.8 (2.4 to 4.8)	3.4 (1.9 to 4.2)	0.89	0.83 to 0.96	0.002
Antiretroviral therapy (ART)					
Never	144 (27.8)	92 (28.2)	1.00		
Ever	375 (72.2)	234 (71.8)	0.98	0.72 to 1.33	0.881
ART in last six months					
0 days	204 (39.3)	130 (39.9)	1.00		
≥1 days	315 (60.7)	196 (60.1)	0.98	0.74 to 1.30	0.869
ART adherence^c^,^d^					
<95%	160 (42.7)	127 (54.3)	1.00		
≥95%	215 (57.3)	107 (45.7)	0.63	0.45 to 0.87	0.005
HIV-1 RNA viral load					
≥50 copies/mL	346 (66.7)	233 (71.5)	1.00		
<50 copies/mL	173 (33.3)	93 (28.5)	0.80	0.59 to 1.08	0.143

a Odds ratio

b 95% confidence interval

c refers to 180-day period prior to the baseline interview

d among 609 baseline ART-exposed individuals.

**Figure 1:**
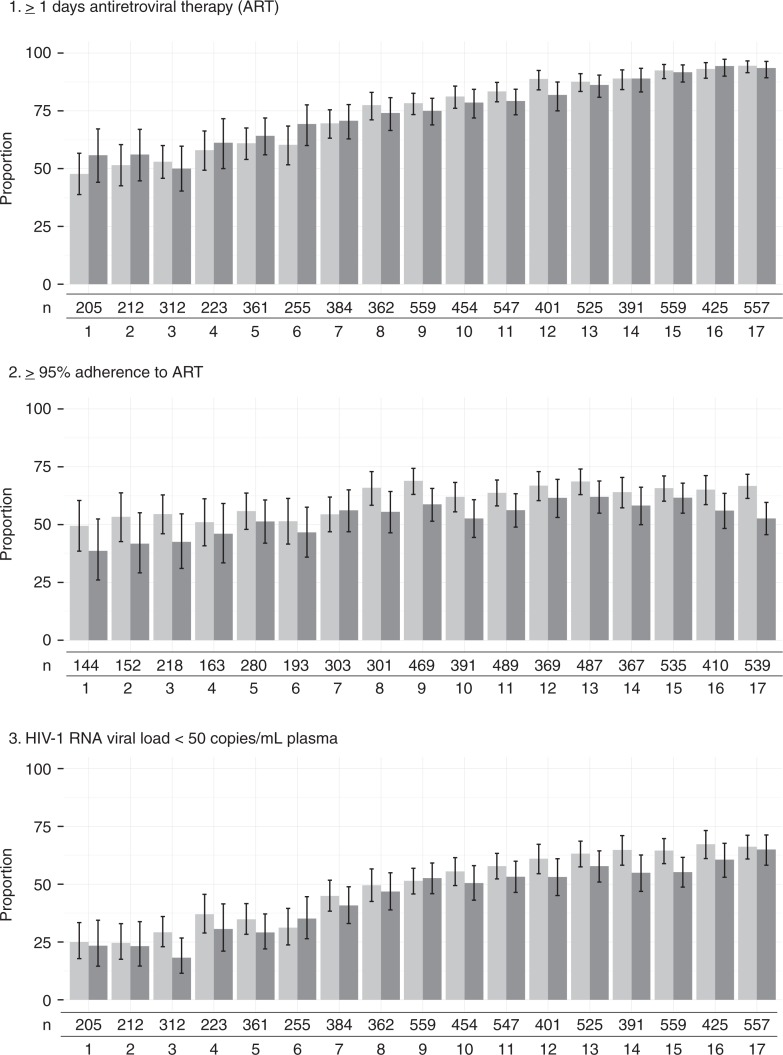
HIV/AIDS treatment outcomes, 2005 to 2014. Proportion, with 95% confidence interval, of participants with ≥1 day of ART in the previous six months (top); ≥95% adherence to ART (middle); and plasma HIV-1 RNA load <50 copies/mL (bottom) stratified by indigenous (dark grey) and non-indigenous (light grey) ancestry.

Figure 1 depicts the changes over time in the proportion of participants experiencing each HIV treatment threshold. At the first interview period, 51% of all participants had been dispensed ≥1 day of ART in the previous 180 days; at the final interview period, 94% had been recently dispensed ART. Similarly, the proportion of all participants attaining ≥95% adherence in the previous 180 days was 45% at the first interview period; at the final interview period, the proportion was 61%. Finally, 56% of all individuals had non-detectable plasma VL at the final interview period, an increase of 32 percentage points since the first interview period.

The results of the longitudinal analyses of difference between indigenous and non-indigenous participants are presented in Table 2. No difference was detected in the likelihoods of indigenous and non-indigenous participants being dispensed ART in the previous 180 days in unadjusted analyses (odds ratio (OR)=0.99, 95% confidence interval (95% CI)=0.77 to 1.27) or analyses adjusted for gender, age, CD4+ cell count and interview period (adjusted odds ratio (AOR)=1.32, 95% CI: 0.92 to 1.89). Although indigenous participants were less likely to attain ≥95% adherence in the last six months over the study period (OR=0.73, 95% CI: 0.63 to 0.89), this difference was not apparent in a model adjusted for socio-demographic and clinical characteristics (AOR=0.90, 95% CI: 0.73 to 1.10). Finally, indigenous participants were as likely as non-indigenous participants to have non-detectable plasma VL in both crude (OR=0.85, 9% CI: 0.70 to 1.05) and adjusted (AOR=1.15, 95%: 0.91 to 1.46) analyses. Notably, the interview period was positively associated with non-detectable VL (AOR=1.09 per interview period, 95% CI: 1.07 to 1.11). In the light of the crude associations between gender and each outcome of interest in Table 2, we conducted analyses to test for interaction between gender and indigenous ancestry. The interaction term in the analysis of recent ART dispensation approached statistical significance (*p*=0.057); the interaction terms in the analyses of ART adherence and non-detectable VL were not significant (*p*>0.5).

**Table 2:** Crude and adjusted longitudinal estimates of likelihood of optimal HIV treatment indicators among 845 indigenous and non-indigenous people who use illicit drugs in Vancouver, Canada

Characteristics	OR^a^	95% CI^b^	*p*	AOR^c^	95%	*p*
**≥1 day of ART in previous 180 days**						
Indigenous ancestry (yes vs. no)	0.99	0.77 to 1.27	0.911	1.32	0.92 to 1.89	0.130
Interview period (per period)	1.19	1.17 to 1.22	<0.001	1.16	1.14 to 1.19	<0.001
Gender (male vs. non-male)	1.60	1.25 to 2.06	<0.001	1.20	0.84 to 1.71	0.312
Age (per year)^d^	1.16	1.14 to 1.19	<0.001	1.07	1.05 to 1.10	<0.001
CD4 cells/mL (per 100 cells)^e^	1.19	1.12 to 1.27	<0.001	1.08	1.03 to 1.15	0.04
**≥95% ART adherence in previous 180 days**^f^						
Indigenous ancestry (Y v N)	0.73	0.63 to 0.89	0.002	0.90	0.73 to 1.10	0.307
Interview period (per period)	1.04	1.03 to 1.06	<0.001	1.01	1.00 to 1.03	0.092
Gender (male vs. non-male)	1.32	1.08 to 1.62	0.008	1.12	0.90 to 1.38	0.300
Age (per year)^d^	1.05	1.03 to 1.06	<0.001	1.03	1.02 to 1.05	<0.001
CD4 cells/mL (per 100 cells)^e^	1.22	1.16 to 1.28	<0.001	1.19	1.14 to 1.26	<0.001
**Plasma HIV-1 RNA viral load<50 copies/mL**						
Indigenous ancestry (Y v N)	0.85	0.70 to 1.05	0.128	1.15	0.91 to 1.46	0.252
Interview period (per period)	1.14	1.12 to 1.16	<0.001	1.09	1.07 to 1.11	<0.001
Gender (male vs. non-male)	1.31	1.07 to 1.62	0.010	1.08	0.84 to 1.38	0.541
Age (per year)^d^	1.12	1.10 to 1.14	<0.001	1.06	1.05 to 1.08	<0.001
CD4 cells/mL (per 100 cells)^e^	1.44	1.36 to 1.53	<0.001	1.37	1.29 to 1.45	<0.001

a Odds Ratio

b 95% confidence interval

c adjusted odds ratio

d at baseline

e time-updated

f among 723 ART-exposed individuals.

Table 3 presents the results of the longitudinal analyses of engagement in different stages of HIV treatment among indigenous participants. The crude (OR=1.18, 95% CI: 1.15 to 1.22) and adjusted (AOR=1.16, 95% CI: 1.11 to 1.20) results reflect significant increases in the proportion of indigenous participants with recent ART dispensation. While the unadjusted analysis reported an increasing proportion of indigenous participants achieving optimal ART adherence over time (OR=1.05, 95% CI: 1.02 to 1.07), there were no significant improvements over time in the multivariable model (AOR=1.01, 95% CI: 0.99 to 1.04). However, significant increases in the proportion of participants with non-detectable VL were observed in bivariable (OR=1.12, 95% CI: 1.09 to 1.15) and multivariable (AOR=1.07, 95% CI: 1.04 to 1.10) models.

**Table 3:** Crude and adjusted longitudinal estimates of likelihood of optimal HIV/AIDS treatment indicators among 326 indigenous people who use illicit drugs in Vancouver, Canada

Characteristics	OR^a^	95% CI^b^	*p*	AOR^c^	95%	*p*
≥**1 day of ART in previous 180 days**						
Interview period (per period)	1.18	1.15 to 1.22	<0.001	1.16	1.11 to 1.20	<0.001
Gender (male vs. non-male)	1.71	1.15 to 2.54	0.008	1.04	0.61 to 1.78	0.882
Age (per year)^d^	1.16	1.12 to 1.20	<0.001	1.06	1.02 to 1.09	0.001
CD4 cells/mL (per 100 cells)^e^	1.14	1.04 to 1.25	0.07	1.02	0.93 to 1.11	0.688
≥**95% ART adherence in previous 180 days**^f^						
Interview period (per period)	1.05	1.02 to 1.07	<0.001	1.01	0.99 to 1.04	0.233
Gender (Male vs. non-male)	1.37	1.02 to 1.84	0.039	1.15	0.85 to 1.57	0.361
Age (per year)^d^	1.05	1.03 to 1.07	<0.001	1.03	1.01 to 1.05	0.001
CD4 cells/mL (per 100 cells)^e^	1.22	1.13 to 1.32	<0.001	1.20	1.10 to 1.29	<0.001
**Plasma HIV-1 RNA viral load <50 copies/mL**						
Interview period (per period)	1.12	1.09 to 1.15	<0.001	1.07	1.04 to 1.10	<0.001
Gender (Male vs. non-male)	1.61	1.17 to 2.23	0.004	1.30	0.89 to 1.90	0.168
Age (per year)^d^	1.13	1.10 to 1.17	<0.001	1.07	1.05 to 1.10	<0.001
CD4 cells/mL (per 100 cells)^e^	1.47	1.32 to 1.63	<0.001	1.39	1.25 to 1.56	<0.001

a Odds ratio

b 95% confidence interval

c adjusted odds ratio

d at baseline

e time-updated

f among 297 ART-exposed individuals.

## Discussion

In this study, we observed significant and substantial increases over time in the proportion of IPLWH who use illicit drugs achieving important thresholds in HIV treatment and care. An increasing proportion of indigenous participants were engaged in care, reflected by recent dispensation of ART, increasing from 56 to 93% during the study. In addition, almost two-thirds of indigenous participants achieved non-detectable VL by the end of the study, compared with less than one-quarter at study start (23 vs. 65%). These changes were observed in separate longitudinal multivariable models adjusted for gender, age and CD4 cell count. Both the magnitude of the improvements and their rate of change were indistinguishable between indigenous and non-indigenous participants.

These findings stand in sharp contrast to previous studies from the earlier era of HIV treatment in this setting, and others which have described important disparities in ART access and outcomes between indigenous and non-indigenous individuals [15,23–31]. Most notably, in a study that considered the period from 1996 to 2003, 53% lower rates of ART initiation were observed among IPLWH who inject drugs in this setting [30]. In a separate study that considered the period from 1995 to 2001 and was not restricted to drug-using individuals, IPLWH in British Columbia were significantly more likely to die before initiating ART compared with non-indigenous PLWHA (45% vs. 31%, *p*=0.001) [31]. Similar disparities have been described in other studies of HIV treatment outcomes in Canada [15,23,24,26,28,29,32]. For example, among 548 HIV-positive patients starting ART in Alberta, Aboriginal ethnicity was associated with higher rates of both all-cause and HIV-related death over follow-up [26]. It is important to note that these studies, as well as the current work, do not contain data on the modifiable determinants of health for people living with HIV that might be driving these health outcomes, such as socio-economic impoverishment [29], gender-based violence [32], stigma and discrimination in health care and other settings [32] and contact with the criminal justice system [33]. Thus, future research should seek to identify the social and structural-level factors associated with the achievements documented in the current study.

The substantial decrease observed in the proportion of indigenous individuals with detectable plasma VL in this study has implications not only for disease progression among these individuals but also for patterns of viral transmission. Both randomized clinical trials [3] and observational studies [34] have demonstrated the close link between higher levels of individual- and community-level VL and the incidence of new infections. The current findings are particularly timely given elevated rates of preventable infections among many indigenous peoples in Canada and uncontrolled ongoing HIV outbreaks including substantial proportions of infections among indigenous individuals [12–35]. For example, in Saskatchewan and Manitoba, where individuals reporting First Nations, Métis or Inuit ancestry represent more than 70 and 40%, respectively, of new HIV diagnoses [35]. Two studies from British Columbia have described ongoing HIV/AIDS treatment deficits among indigenous individuals [36,37]. In an analysis of data on individuals with CD4 cell counts <500 cells/mL in a BC-wide treatment registry, individuals with Aboriginal ancestry were statistically significantly less likely to be virologically suppressed [37]. Nevertheless, while these studies describe ongoing HIV treatment deficits among indigenous individuals, we observed substantial improvements in HIV treatment outcomes among indigenous illicit drug users in a major urban setting. Our results are in line with the findings of a recent study from the same setting which described improvements in plasma VL suppression among 269 patients at an urban clinic in this setting and no significant differences between Aboriginal and non-Aboriginal individuals [38].

Our findings have immediate implications for policy and practice. First, while we observed impressive improvements in three key measures of HIV care among a traditionally under-treated group, a substantial proportion of indigenous and non-indigenous individuals continue to experience sub-optimal treatment outcomes. Renewed emphasis should be placed on addressing the social and structural barriers to effective ART, including homelessness [21], high-risk income generation strategies (e.g. sex work and drug dealing) [39] and criminalization [40]. Second, our findings suggest that efforts to scale up HIV treatment access among illicit drug users should be a part of renewed strategies to prevent new HIV infections among indigenous individuals. In Canada, the federal government has constitutional responsibility for delivering health care to indigenous individuals in various parts of the country. It is notable that the Canadian federal government in power from 2006 to 2015 was not only strongly opposed to many evidence-based harm reduction interventions to reduce HIV transmission [41] but also failed to formally adopt TasP as a component of Canada's national HIV/AIDS strategy, which was last updated in 2006 by the previous administration.

This study has limitations. First, there is increasing awareness of the importance of indigenous-led systems to identify indigenous individuals, especially in government and research data, in decolonization and self-government efforts [42]. In addition to the heterogeneity of indigenous individuals and groups across Canada, there are concerns that administrative identifiers may not capture all important aspects of indigeneity, including ancestry, identity and political status [43]. In this study, we relied on participant self-report, recognizing this might overstate or understate important aspects of indigenous status in this setting. Second, although this observational study was composed of illicit drug users recruited from community settings, we cannot conclude that the observed trends in HIV treatment status over time are representative of the larger population of HIV-positive indigenous drug users in Vancouver nor of indigenous people in British Columbia and have no evidence to suggest that indigenous participants were more socially integrated than non-indigenous participants. Fortunately, our analyses benefit from using data from a large cohort of illicit drug users followed over the long term using outcomes ascertained from an administrative source of comprehensive clinical monitoring data. Finally, while our analyses indicated significant and substantial improvements coincident with a province-wide TasP initiative, we did not evaluate potentially causal factors related to these changes, such as exposure to interventions related to the campaign. To help optimize indigenous-focused HIV prevention and treatment campaigns in other settings, future research should endeavour to identify the meaningful social and structural-level components of these programmes. As noted above, this is especially important for many indigenous groups from which leaders, elders and community members have expressed desire for evidence to support health and wellness.

## Conclusions

To conclude, we sought to evaluate longitudinal changes in HIV treatment indicators among indigenous people who use illicit drugs during a community-wide TasP intervention in British Columbia, Canada. Between 2005 and 2013, we observed significant and substantial increases in the proportion of individuals with recent dispensation of ART, achieving optimal adherence to ART and attaining non-detectable levels of plasma VL. These results not only describe impressive clinical achievements by these individuals but also support the immediate scale-up of efforts to engage indigenous individuals in HIV treatment and care as a means to reduce elevated levels of HIV/AIDS-associated morbidity, mortality and viral transmission.
